# Unilateral Left-Hand Contractions Produce Widespread Depression of Cortical Activity after Their Execution

**DOI:** 10.1371/journal.pone.0145867

**Published:** 2015-12-28

**Authors:** Fernando Cross-Villasana, Peter Gröpel, Michael Doppelmayr, Jürgen Beckmann

**Affiliations:** 1 Chair of Sport Psychology, Technische Universität München, München, Germany; 2 Institute of Sport Science, Johannes Gutenberg Universität, Mainz, Germany; University Medical Center Groningen UMCG, NETHERLANDS

## Abstract

The execution of unilateral hand contractions before performance has been reported to produce behavioral aftereffects in various tasks. These effects have been regularly attributed to an induced shift in activation asymmetry to the contralateral hemisphere produced by the contractions. An alternative explanation proposes a generalized state of reduced bilateral cortical activity following unilateral hand contractions. The current experiment contrasted the above explanation models and tested the state of cortical activity after the termination of unilateral hand contractions. Twenty right-handed participants performed hand contractions in two blocks, one for each hand. Using electroencephalogram (EEG), the broad alpha band and its asymmetry between hemispheres before, during, and after hand contractions were analyzed. During contractions, significant bilateral decrease in alpha amplitudes (indicating cortical activation) emerged for both hands around sensory-motor regions. After contractions, alpha amplitudes increased significantly over the whole scalp when compared to baseline, but only for the left hand. No modulation of hemispheric asymmetry was observed at any phase. The results suggest that unilateral hand contractions produce a state of reduced cortical activity after their termination, which is more pronounced if the left hand was used. Consequently, we propose that the reduced cortical activity (and not the persistent activation asymmetry) may facilitate engagement in subsequent behavior, probably due to preventing interference from other, nonessential cortical regions.

## Introduction

The present research tested the changes in the state of cortical activity after the termination of unilateral hand contractions. Unilateral hand contractions, also referred as hand clenching, consist of executing a vigorous grip movement with one hand for a brief period of time (e.g., [1–7]). Numerous studies [1–7] report that unilateral hand contractions produce particular behavioral effects after their execution. These effects have been attributed to an induced shift in activation asymmetry to the contralateral hemisphere induced through the contractions, hence enhancing the functions in which that hemisphere specializes and affecting performance in a subsequent task. Among the reported effects are heightened approach motivation after right contractions [1, 2, 4]; increased processing to global visual stimuli after left, and local stimuli after right contractions [5]; enhanced memory encoding after right, and retrieval after left contractions [3]; and prevention of motor skill failure under pressure after left hand contractions [6]. In the above studies, it is assumed that the activation asymmetry towards the contralateral hemisphere induced through unilateral hand contractions persists even after their termination (referred to hereafter as a *persistent activity model*). However, this notion has not been tested directly and an alternative mechanism is possible.

The persistent activity model suggests that during the hand contractions, activity spreads from the contralateral motor cortex towards adjacent regions in the same hemisphere, producing a one sided increase in basal hemispheric activity which persists after terminating contractions and thus affects subsequent behavior [4, 6, 7]. Electroencephalogram (EEG) studies found a greater relative left activation during the right hand contractions and a greater relative right activation during the left hand contractions [1, 2, 5]. Moreover, this biased activation asymmetry towards the contralateral hemisphere affected not only the motor area, but was also observed over frontal, temporal and parietal locations, supporting the notion that during contractions, activity of the motor cortex recruits other adjacent cortical regions. This overspread activity might thus facilitate subsequent behaviors that rely on the functions of the involved hemisphere [8].

An alternative explanation, referred to hereafter as a *reduced activity model*, can be deduced from experimental findings using Transcranial Magnetic Stimulation (TMS). A long-lasting depression of cortical excitability has been reported after the execution of repetitive unilateral hand movements similar to the hand contractions [9–11]. When using the right hand (lifting a weight with the wrist [9]; abduction-adduction of the thumb [10]), the depression has been observed at the contralateral hemisphere; when using the left hand (pinch grips, [11]) the depression has been observed at both hemispheres. This suggests that while activity is increased in the contralateral hemisphere *during* unilateral hand contractions [1, 2, 5], a state of reduced cortical activity may take place *after* the contractions, which is probably more widespread if the left hand was used [9–11]. Empirical evidence further supports that a tonic reduction in cortical activity, reflected by an increase in the EEG alpha band, implies a state of readiness for processing information [12–15], and preparing motor actions [16–19]. Consequently, the reduced cortical activity might contribute to enhanced cognitive and motor performance by facilitating task-specific cortical activations, and preventing interference from other, nonessential cortical regions [13–17, 20, 21].

Thus, these two explanatory models make different predictions about how unilateral hand contractions affect cortical activity and subsequent task performance. The persistent activity model predicts that the induced dominant activation of the contralateral hemisphere observed during unilateral hand contractions [1, 2, 5] prevails after their termination. The reduced activity model predicts that after the termination of unilateral hand contractions, a generalized state of reduced cortical activity emerges. In addition, based on current findings [9–11], we assume that the left and right hands might differ regarding the strength of the effect. These predictions were tested in the present EEG experiment. In particular, we analyzed the amplitude of the EEG broad alpha band (8–12 Hz) and its asymmetry between hemispheres before, during and after hand contractions. Alpha band power represents the inverse of cortical activation [13, 14, 22] and is known to affect the cortical response to TMS [23–28], therefore reflecting a state similar to that observed in TMS experiments after repetitive hand movements.

Distinguishing between the two hypothesized effects of unilateral contraction is relevant for a better understanding of the mechanisms underlying the reported behavioral aftereffects of hand contractions. Furthermore, if hand contractions induce an increase of alpha amplitudes after their termination, they may represent an additional alternative to other established methods of alpha entrainment like repetitive TMS (rTMS) [29] or transcranial alternating current stimulation (tACS) [22, 30] with their prospective clinical applications.

## Materials and Methods

### Study Design

The study used a within-subjects design in which the effects during and after contractions of each hand on cortical activity were analyzed in comparison to their respective baselines through EEG. Differences between baselines were also analyzed. Only data of right handed subjects were included. In order to examine only the effects produced by unilateral hand contractions on cortical activity, no further tasks were tested. To prevent the experiment from getting too long, hence to avoid possible confounds of tiredness or boredom on the EEG, only one single series of repetitive hand contractions was carried for each hand. Note that in a previous study by our research group a single series of contractions sufficed for producing subsequent behavioral effects [6].

### Participants

Twenty five voluntary participants were recruited for the experiment. Three of the participants were not included for analysis due to technical errors. One participant was left handed as indicated by the Edinburgh Handedness Inventory [31] and therefore, her data were removed. Another participant had to abandon the experiment owing to breathing problems due to a spring allergy. The final sample consisted of 20 right-handed participants (11 female), with a mean age of 22.9 years (range: 19 to 26 years). Their mean laterality quotient was +82.23 (range: +52.94 to +100). Prior to entering the study, all participants were informed of the procedures, assured the right to quit the experiment at any moment with no consequences, and asked to sign an informed consent according to the Declaration of Helsinki. The study did not concern medical research neither involved any invasive or potentially dangerous methods for participants and, in accordance with the German Science Foundation, formal approval was not required. At the Technishe Universität München, behavioral sciences are a relatively new area and an ethics committee is in process of being established. At present, projects are presented in a colloquium in which ethical considerations are also discussed. The current research has found approval in such a colloquium.

### Task and Apparatus

Participants sat comfortably on a padded chair in a silent, ample room with attenuated light. The hand contraction task consisted in holding a soft rubber ball (6 cm diameter) in one hand and squeezing it completely with all fingers at a self-pace towards an approximate rate of two times a second, for 45 seconds, while keeping the other hand over their lap with the palm facing down. A small camera outside participants’ view allowed the experimenter to verify correct task performance. To reduce eye movements, participants were requested to look at a grey fixation cross against a black background, presented on a computer screen. To facilitate fixation, the cross flickered from gray to white for 10 msec at variable intervals of 2, 4 or 6 seconds. The same flicker was used for all experimental conditions.

### Procedure

Participants were informed that the experiment tests brain activity patterns elicited by hand movements. After signing an informed consent, participants completed the Edinburgh Handedness Inventory [31]. The scale consists of 10 items where participants rate their preference regarding hand use in various activities such as writing, drawing, throwing, or using scissors. Laterality coefficients range from -100 to +100, and a person is considered to be right-handed when his or her value is higher than +50. Before EEG preparation, participants were instructed how to execute the hand contractions, given an example, and were requested to demonstrate correct understanding. The procedure was shown by the experimenter once again before starting the recording. EEG recording was carried out in two blocks, one for each hand (hand-blocks). Each hand-block consisted of three phases: a two minute resting baseline, 45 seconds of hand contractions and two minutes resting after contractions. After completing one hand-block, a two minute break followed in which the participant could stretch, in order to prevent carry over effects before the next measurement. Before resuming recording, electrode impedances were checked again. Next, a new block was recorded for the other hand. The initial hand-block was counter-balanced. In order to reduce effects of preparation of movement over brain activity, during all resting recordings, the rubber ball stayed at reach behind the participants, outside visual range. For every baseline, the participants were informed in advance which hand was going to follow.

### EEG recording and pre-processing

EEG was recorded with a 64 Ag/AgCl active electrode actiCAP system (Brain Products, Munich, Germany), over an elastic cap (Easy Cap, FMS), with electrodes placed according to the international 10/10 system [32]. All electrodes were referenced to position FCz, and afterwards offline re-referenced to linked mastoids (TP9, TP10). The ground electrode was placed at location AFz. Vertical electrooculogram (VEOG) was registered from an electrode placed beneath the left eye and Fp1. Data was recorded with a Brain Amp amplifier (Brain Products, Munich, Germany), using a band-pass filter from 0.1 to 250 Hz and a notch filter set at 50 Hz, with a sampling rate of 1000 Hz. All electrode impedances were kept under 3 kΩ to prevent large impedance differences between homologous sites. Before EEG analysis, all data was visually inspected to remove large artifacts, re-sampled to a power of two (1024 Hz) and filtered using an infinite-impulse-response (IIR) filter as implemented in Brain Vision Analizer 2 [33] with a high pass of 0.5 Hz, a low pass of 40 Hz and 24 dB slope, notch filter enabled at 50 Hz. An infomax independent component analysis (ICA) as implemented in Brain Vision Analyzer 2 [34, 35] was run and high energy components that reflected eye movements were subtracted.

Continuous EEG recordings were segmented into 2 sec contiguous epochs with 50% overlap for the phases before, during and after contractions of each hand. Automatic artifact rejection was performed on each epoch with a maximum allowed amplitude of ±100 μV, maximum allowed voltage steps of 50 μV between two sampling points, and a minimum required signal change of 0.5 μV in 500 ms. Spectral amplitudes were extracted through a fast Fourier transformation using a Hamming Window with a 50% overlap between contiguous epochs, leading to a spectral resolution of 0.5 Hz. All the 2 sec artifact free epochs within each phase were averaged together. Based on Harmon Jones [1] and Peterson et al. [2], we extracted amplitudes of the whole range of the broad alpha band (8–12 Hz) for homologous electrode pairs Fp1-Fp2, F3-F4, F7-F8, C3-C4, FC3-FC4, FT7-FT8, CP3-CP4, T7-T8, P7-P8, P3-P4, O1-O2 of the 10/10 system, and analyzed the obtained amplitudes and their asymmetry ratio ([right-left/right+left]; [36]).

### Data Analyses

Alpha amplitudes of single electrodes were submitted to a repeated measures ANOVA with the factors “electrode” (Fp1, Fp2, F3, F4, F7, F8, C3, C4, FC3, FC4, FT7, FT8, CP3, CP4, T7, T8, P7, P8, P3, P4, O1, O2), “hand-block” (left, right), and “phase” (before, during and after contractions).

Likewise, the obtained asymmetry-ratios from paired homologue electrodes were submitted to a repeated measures ANOVA with the factors “pair” (Fp1-Fp2, F3-F4, F7-F8, C3-C4, FC3-FC4, FT7-FT8, CP3-CP4, T7-T8, P7-P8, P3-P4, O1-O2), “hand-block” (left, right) and “phase” (before, during and after contractions). Greenhouse-Geisser values are reported when necessary. All significant results from the analyses were post-hoc analyzed with pairwise *t*-tests, using Bonferroni adjusted alpha levels, and their effect sizes assessed with Cohen’s *d*_*z*_ [37]. To confirm analyses, all EEG data was further re-processed with an Average-Reference, and with a reference-free surface-Laplacian [38–42], and the statistical procedures were repeated. For all analyses, an interaction of the factors “hand-block” and “phase” would indicate that hand contractions modulated the alpha amplitudes or asymmetry ratios across the measuring phases.

To gain further insight, we also explored the cortical reaction immediately following hand contractions. Planned comparisons based on previous literature [43–46] were implemented using the broad (8–12 Hz) and upper (10–12 Hz) alpha bands. In these comparisons, the grand-average amplitudes at the first second of the post-contraction period, were compared with the last second of the contraction period [44, 45], and with the last three seconds post-contractions. Fourier transforms over epochs of one single second led to a spectral resolution of 1 Hz. In order to explore the spread of these effects over the scalp, we analyzed electrodes C3, C4, O1, O2 [44, 47], as well as frontal locations F3 and F4 [25, 40, 41, 48].

## Results

### Alpha amplitudes

The repeated measures ANOVA revealed a significant main effect of phase, *F*(2, 38) = 20.36, *p* = .001, $\eta_{p}^{2}$ = .52. Post-hoc t-tests corrected for 3 comparisons, with all scalp electrodes averaged for each phase with both hands, indicated that regardless of the hand-block, greater alpha amplitudes were observed before contractions (*M* = 1.91, *SD* = .60) than during contractions (*M* = 1.76, *SD* = .54), *t*(19) = 2.94, *p* = .02, *d*_*z*_ = .74, and weaker than after contractions (*M* = 2.07, *SD* = .70), *t*(19) = -4.10, *p* = .002, *d*_*z*_ = .92. Consequently, weaker alpha amplitudes were observed during contractions than after contractions, *t*(19) = -5.67, *p* = .001, *d*_*z*_ = 1.27. The analysis further revealed a significant main effect of electrode, *F*(21, 399) = 22.67, *p* = .001, $\eta_{p}^{2}$ = .58, which merely implies amplitude differences among various electrodes to others throughout the whole experiment. There was no main effect of hand-block, indicating that the average alpha amplitudes of the two hand-blocks across all recording phases did not differ significantly.

**Fig 1:**
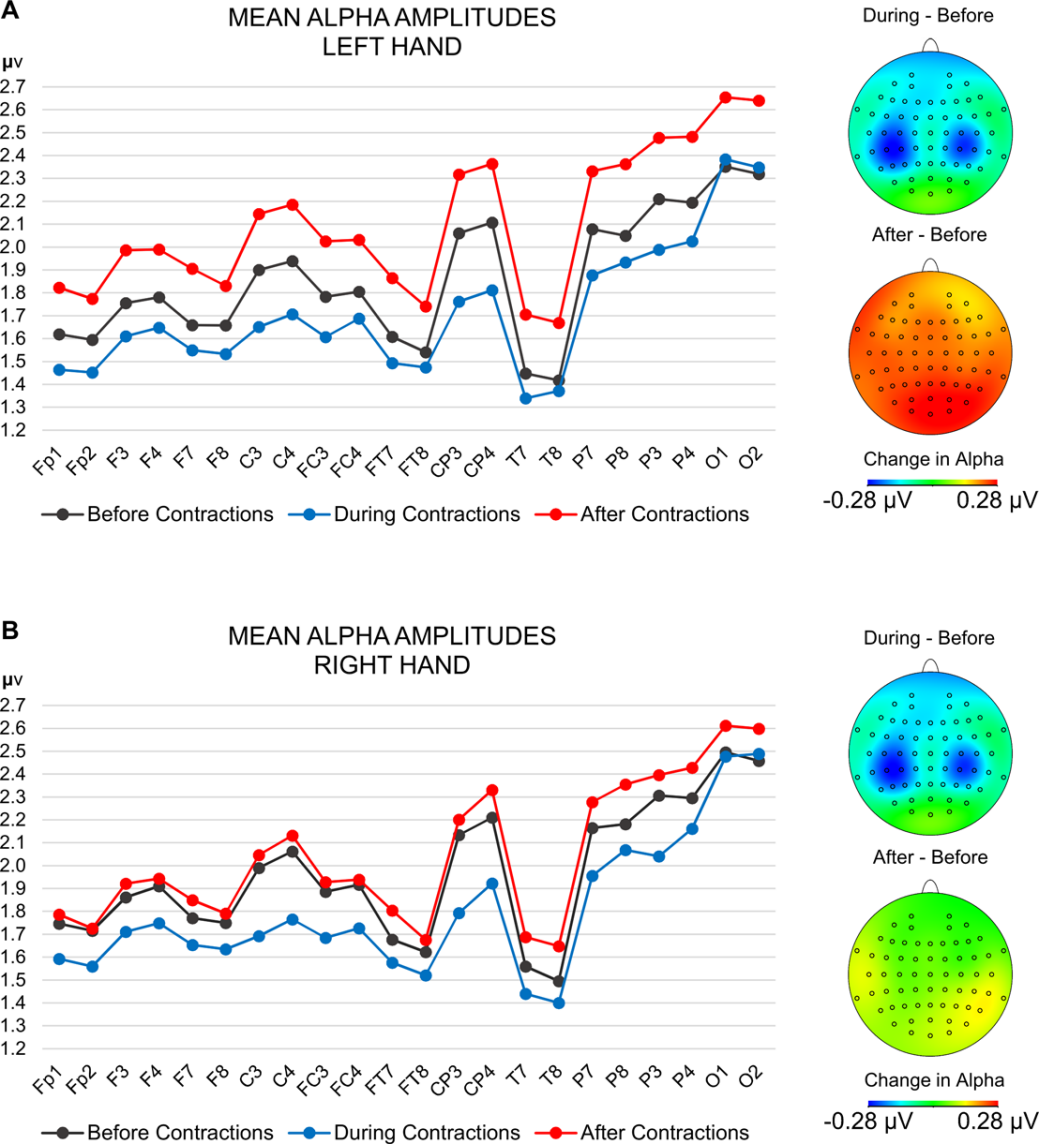
Plot of the alpha amplitudes at each electrode before, during and after contractions. A) For the left hand-block. B) For the right hand-block. Accompanying difference maps indicate the distribution of amplitude changes on the scalp when subtracting the baseline before contractions from the phases during and after contractions.

Our main interest was in how cortical activity changes during and after contractions as a function of the hand used. The analysis revealed a significant interaction of phase and hand-block, *F*(2, 38) = 3.47, *p* = .04, $\eta_{p}^{2}$ = .15, which indicates that amplitude changes within each electrode at each phase were modulated by the hand used for contractions. Table 1 shows alpha amplitudes for all analyzed single electrodes during the two hand-blocks. Post-hoc *t*-tests comparing the values within each electrode across phases for each hand separately are summarized in Table 2. During contractions, significant bilateral decreases in alpha amplitudes took place for both hands around sensory-motor regions, followed by a marked increase after contractions which, compared to baseline, was statistically significant only for the left hand at all electrode locations except F8 and O1. Fig 1 plots alpha amplitudes in every electrode for each hand-block at all phases. Accompanying difference maps display the subtraction of alpha amplitudes of the baselines from their respective periods during and after contractions of each hand. Bilateral decreases in alpha can be observed during contractions for both hands, while after contractions, a generalized increase in alpha amplitude is evident only for the left hand. The right hand shows a smaller and more focal alpha-increase which only reached statistical significance at electrodes P8 and O2.

**Table 1:** 

Electrode Position	Left Hand Contractions			Right Hand Contractions		
	Before	During	After	Before	During	After
Fp1	1.61 (.48)	1.46 (.46)	1.82 (.65)	1.75 (.59)	1.59 (.51)	1.79 (.61)
Fp2	1.59 (.47)	1.45 (.40)	1.78 (.62)	1.72 (.54)	1.56 (.47)	1.73 (.56)
F3	1.76 (.59)	1.61 (.52)	1.99 (.74)	1.86 (.67)	1.71 (.61)	1.92 (.70)
F4	1.78 (.55)	1.64 (.47)	1.99 (.71)	1.91 (.64)	1.75 (.57)	1.94 (.66)
F7	1.66 (.54)	1.55 (.48)	1.91 (.76)	1.77 (.65)	1.65 (.62)	1.85 (.67)
F8	1.66 (.50)	1.53 (.43)	1.83 (.67)	1.75 (.60)	1.63 (.56)	1.79 (.60)
C3	1.90 (.61)	1.65 (.43)	2.14 (.77)	1.99 (.66)	1.69 (.50)	2.05 (.70)
C4	1.94 (.64)	1.71 (.48)	2.19 (.75)	2.06 (.66)	1.76 (.54)	2.13 (.69)
FC3	1.78 (.57)	1.61 (.46)	2.02 (.73)	1.89 (.62)	1.68 (.55)	1.93 (.65)
FC4	1.81 (.54)	1.69 (.47)	2.03 (.68)	1.92 (.60)	1.73 (.52)	1.94 (.59)
FT7	1.61 (.57)	1.49(.45)	1.86(.73)	1.68(.63)	1.57(.58)	1.80(.63)
FT8	1.54 (.54)	1.47 (.42)	1.74 (.66)	1.62 (.60)	1.52 (.57)	1.67 (.63)
CP3	2.06 (.65)	1.76 (.44)	2.31 (.82)	2.13 (.66)	1.79 (.49)	2.20 (.70)
CP4	2.11 (.75)	1.81 (.52)	2.36 (.85)	2.21 (.71)	1.92 (.60)	2.33 (.80)
T7	1.45 (.59)	1.34 (.47)	1.71 (.75)	1.56 (.55)	1.44 (.50)	1.69 (.65)
T8	1.41 (.62)	1.37 (.50)	1.67 (.77)	1.50 (.65)	1.40 (.60)	1.65 (.70)
P7	2.08 (.73)	1.88 (.63)	2.33 (.94)	2.16 (.75)	1.95 (.69)	2.28 (.83)
P8	2.05 (.73)	1.93 (.73)	2.36 (.91)	2.18 (.74)	2.07 (.82)	2.35 (.85)
P3	2.21 (.74)	1.99 (.62)	2.48 (.94)	2.31 (.75)	2.04 (.61)	2.40 (.81)
P4	2.19 (.74)	2.02 (.69)	2.48 (.90)	2.30 (.72)	2.16 (.75)	2.43 (.81)
O1	2.35 (.78)	2.38 (1.02)	2.65 (1.10)	2.49 (.96)	2.47 (1.07)	2.61 (1.00)
O2	2.31 (.81)	2.34 (1.02)	2.64 (1.10)	2.46 (.93)	2.49 (1.08)	2.60 (1.02)

Mean (*SD*) alpha amplitudes for each electrode and each hand across the phases.

**Table 2:** 

Electrode Position	Left Hand Contractions				Right Hand Contractions			
	Before vs. During		Before vs. After		Before vs. During		Before vs. After	
	*t*(19)	*d*_*z*_	*t*(19)	*d*_*z*_	*t*(19)	*d*_*z*_	*t*(19)	*d*_*z*_
Fp1	2.71*	.61	-3.53**	.79	2.19	.49	-1.23	.28
Fp2	2.45	.55	-3.31**	.74	2.44	.55	-2.82	.63
F3	2.67*	.60	-3.97**	.89	2.38	.53	-1.72	.38
F4	2.27	.51	-3.56**	.80	2.58	.58	-1.11	.25
F7	2.16	.48	-3.26*	.73	1.66	.37	-2.15	.48
F8	1.76	.39	-2.53	.57	1.55	.35	-1.11	.25
C3	3.98**	.89	-3.30*	.74	3.76**	.84	-.91	.20
C4	3.45**	.77	-4.37**	.98	3.87**	.87	-1.38	.31
FC3	2.83*	.63	-3.51**	.79	3.37*	.75	-.87	.19
FC4	1.93	.43	-3.83**	.86	3.13*	.70	-.52	.12
FT7	2.07	.46	-3.70**	.83	1.75	.39	-2.02	.45
FT8	1.13	.25	-3.82**	.85	1.37	.31	-.998	.22
CP3	4.52**	1.01	-3.64**	.81	4.17**	.93	-.909	.20
CP4	3.86**	.86	-4.65**	1.04	4.55**	1.02	-2.23	.50
T7	1.94	.43	-3.38**	.76	1.92	.43	-1.68	.38
T8	.64	.14	-3.60**	.81	1.34	.30	-2.45	.55
P7	2.55	.57	-3.26**	.73	3.07	.69	-2.14	.48
P8	1.01	.23	-3.59**	.80	1.39	.31	-3.74**	.84
P3	2.83*	.63	-3.76**	.84	3.88**	.87	-1.30	.29
P4	1.81	.40	-4.38**	.98	2.14	.48	-2.58	.58
O1	-.023	.01	-2.73	.61	.186	.04	-2.55	.57
O2	-.024	.01	-3.52**	.79	-.338	.08	-3.12*	.70

*t*-scores and effect sizes for differences in alpha amplitudes between the phases before and during and the phases before and after hand contractions for each electrode and each hand.

*indicates significance *p* < .016, and

**indicates significance *p* < .003 (corrected for multiple (3) comparisons–Bonferroni).

A comparison of the baselines before left and right contractions revealed significant differences at frontal electrodes (S1 Table), with higher alpha amplitudes before right hand contractions. A closer inspection revealed that in those participants which first performed the left hand-block, the increase in alpha amplitudes prevailed through the baseline before right-contractions (S1 Fig). Therefore, to certify that the observed increase of alpha amplitudes only after left contractions was not confounded by the order of the hand-blocks, a complementary analysis was implemented. The sample was split into right-first and left-first sub-groups, and *t*-tests comparing alpha values before and after contractions were repeated for each sub-group. Given the smaller size of the sub-groups, Bonferroni correction was not considered. This analysis confirmed that in each sub-group, while the left hand produced significant increases in alpha amplitudes after contractions at most electrodes (S2 Table), the right hand produced smaller increases in fewer electrodes. In sum, these results support the reduced activity model. Cortical activity declined after the termination of hand contractions, this effect was however more pronounced for the left hand.

A further interaction was observed between electrode and phase, *F*(42, 798) = 4.49, *p* = .002, $\eta_{p}^{2}$ = .19, indicating that amplitude differences among electrodes varied with each phase. Post-hoc analysis displayed in the supplementary material (S1 Annex, S2 Fig) revealed that occipital and central electrodes increased the difference between their amplitudes during hand contractions of any hand. This owes to the fact that during contractions, occipital electrodes slightly, albeit non-significantly, increased their alpha amplitudes (Table 1), while central electrodes showed the greatest decrease. This observation is in line with classical findings in the literature where hand movements produced a simultaneous activation on motor regions over the scalp, and inhibition over visual regions [47].

No interaction of electrode and hand-block was observed, indicating that the differences between electrodes were not affected by the hand used. Finally, the three-way interaction of hand-block, electrode and phase was not significant. In the confirmation analysis with alternative EEG referencing schemes (S2 Annex) the Surface Laplacian corroborated the currently observed effects, namely, an interaction between phase and hand-block *F*(2, 38) = 3.29, *p* = .048, $\eta_{p}^{2}$ = .15, while the average reference showed the same patterns, albeit the aforementioned interaction did not reach statistical significance *F*(2, 38) = 2.43, *p* = .101, $\eta_{p}^{2}$ = .11.

### Alpha asymmetry ratios

Tests for asymmetry ratios showed any main effects neither of hand-block, pair, phase or their interactions. The lack of interaction between the factors phase and hand-block, *F*(2, 38) = .43, *p* = .65 $\eta_{p}^{2}$ = .02, indicates that hand contractions did not induce systematic modulations of asymmetry within each electrode pair. This lack of effect can be visualized in Fig 1, where despite of overall amplitude changes at each phase, the amplitude ratio between homologue electrodes (i.e., F3-F4, C3-C4, etc.) remains mostly constant and is not systematically modified by left or right hand contractions. Thus, we could not replicate the findings of Harmon-Jones [1] and Peterson et al. [2] who reported greater relative activation in electrodes contralateral to the active hand. Consequently, our results are contrary to the persistent activity model.

### Cortical reaction immediately following contractions

**Fig 2:**
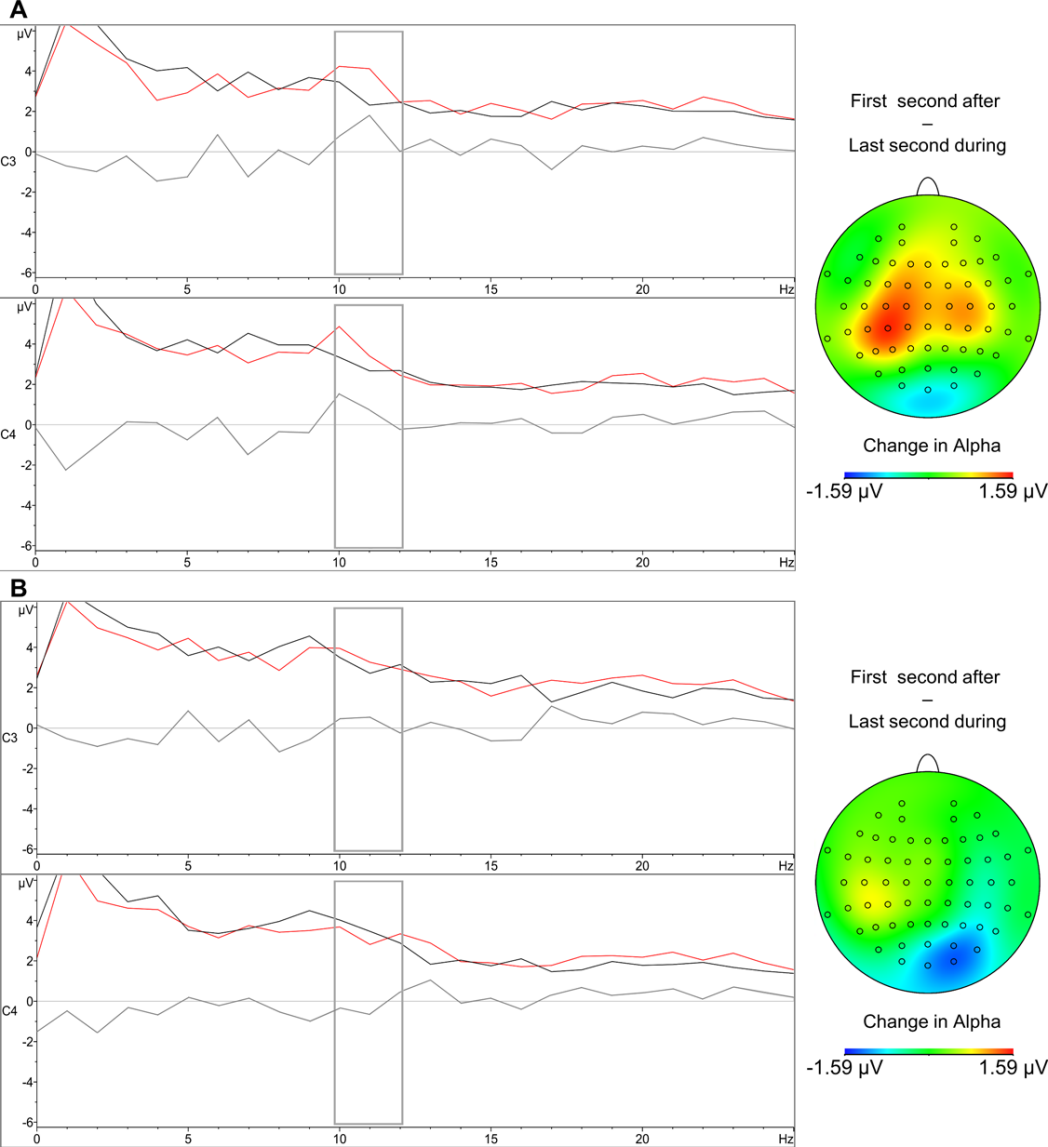
Spectral plot of the last second after contractions (black), first second after contractions (red), and their difference (gray). A) For the left hand-block. B) For the right hand-block. The gray rectangle highlights the upper alpha band. Accompanying difference maps illustrate the difference over the scalp when subtracting the last second of contractions, from the first second after contractions.

Explorative comparisons based on previous literature [43–46] revealed a bilateral amplitude increase restricted to the upper alpha range (10–12 Hz) over motor regions immediately following left hand and to a lesser degree, right hand contractions (Fig 2). In the first second after left contractions this increment was significant on electrodes C3 (*M* = 4.18 μV, *SD* = 2.39) and C4 (*M* = 4.14 μV, *SD* = 1.85) compared with their respective last second during contractions in C3 (*M* = 2.89 μV, *SD* = 1.23) *t*(19) = 2.48, *p* < .05, *d*_*z*_ = .55 and C4 (*M* = 3.01 μV, *SD* = 1.21), *t*(19) = 3.03, *p* < .01, *d*_*z*_ = .68. An accompanying peak was observed at 22 Hz over C3 (Fig 2) after termination (*M* = 2.71 μV, *SD* = 1.35) over the last contraction second (*M* = 2.01 μV, *SD* = 1.17) with marginal significance *t*(19) = 1.98, *p* = .06, *d*_*z*_ = .44, which likely represents a harmonic component of the upper alpha enhancement [44]. These observations coincide with previously reported rebounds of the Mu rhythm, an inhibitory rhythm of the motor cortex which overlaps with the upper range of alpha [44, 48] after single hand movements [44, 46].

**Fig 3:**
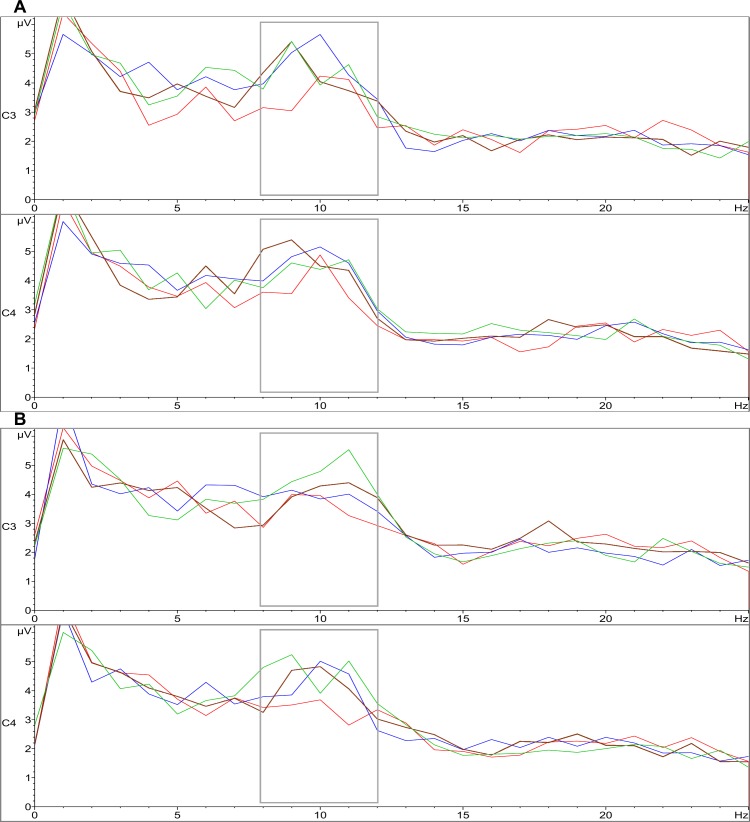
Spectral plot of the first (red), antepenultimate (blue), penultimate (brown) and last (green) seconds after contractions. A) For the left hand-block. B) For the right hand-block. The gray rectangle highlights the broad alpha band.

When comparing upper alpha at C3 and C4 in the first second after left contractions with the last three seconds of the same period, no significant differences were found. This is given that the already enhanced upper alpha at the first second covers part of the broad alpha band (8–12 Hz), which also showed enhancements in the last seconds (Fig 3). Therefore, if the former comparison is carried using broad alpha, the antepenultimate and penultimate seconds show a significant amplitude increase, although the last second fails to reach significance (Table 3).

**Table 3:** 

**Broad alpha amplitude after contractions: Left hand**										
	Firstsecond	AntepenultimateSecond			PenultimateSecond			Lastsecond		
	**Mean**	**Mean**	***t*(19)**	***d***_***z***_	**Mean**	***t*(19)**	***d***_***z***_	**Mean**	***t*(19)**	***d***_***z***_
C3	3.64	4.74	-3.15**	.70	4.39	-2.44*	.55	4.44	-1.62	.36
C4	3.86	4.64	-2.18*	.49	4.83	-2.77*	.62	4.36	-1.15	.26
F3	3.40	4.18	-1.57	.35	3.92	-1.42	.32	4.30	-1.87	.42
F4	3.28	4.41	-3.01**	.67	4.01	-1.99	.44	4.33	-2.11*	.47
O1	3.95	6.21	-3.99**	.89	6.12	-3.31**	.74	5.25	-2.62*	.59
O2	4.24	5.54	-3.10**	.69	6.53	-3.34**	.75	5.52	-2.01	.45
**Broad alpha amplitude after contractions: Right hand**										
	Firstsecond	AntepenultimateSecond			Penultimatesecond			LastSecond		
	**Mean**	**Mean**	***t*(19)**	***d***_***z***_	**Mean**	***t*(19)**	***d***_***z***_	**Mean**	***t*(19)**	***d***_***z***_
C3	3.52	3.98	-1.16	.26	3.88	-1.04	.23	4.65	-2.71*	.61
C4	3.36	4.31	-2.10*	.47	4.21	-1.58	.35	4.75	-3.04*	.68
F3	3.59	3.60	-.01	0	3.69	-.27	.06	4.50	-2.50*	.56
F4	3.56	3.63	-.25	.06	3.80	-.66	.15	4.44	-2.17*	.49
O1	4.21	5.57	-2.39*	.53	5.47	-2.80*	.63	5.75	-2.92**	.65
O2	4.25	5.48	-2.90**	.65	6.02	-4.42**	.99	5.32	-2.17*	.49

*t*-scores and effect sizes for differences in alpha amplitudes between the first second after contractions and the last three seconds of the same period.

*indicates significance *p* < .05, and

**indicates significance *p* < .01.

With the right hand, a contralateral rebound of upper alpha was observed at C3 (Fig 3), but did not reach statistical significance, no ipsilateral rebound at C4 was observed (Fig 3). This suggests that although a unilateral rebound of the Mu rhythm was observable after right contractions as in previous reports [43; 44], it had lesser intensity, and could not exceed the statistical threshold on the present setting (only one trial per participant). When comparing upper alpha in the first second after contractions at C3 (*M* = 3.61 μV, *SD* = 2.10) and C4 (*M* = 3.25 μV, *SD* = 1.46) versus the respective last three seconds, a significant enhancement is observed in the last second at C3 (*M* = 5.16 μV, *SD* = 2.87), *t*(19) = -2.53, *p* < .05, *d*_*z*_ = .57 and C4 (*M* = 4.47 μV, *SD* = 2.43), *t*(19) = -2.10, *p* = .05, *d*_*z*_ = .47, owing to increases in the broader alpha band at the end (Fig 3). Therefore, when using broad alpha for the same comparison, a significant increase is observed for the last second for both at C3 and C4 (Table 3), and at the antepenultimate second over C4 only (Table 3).

Frontal and occipital electrodes included in this analysis did not show significant changes in upper or broad alpha immediately following hand contractions. However, when comparing the first second versus the last three seconds after contractions, most electrodes showed increases in broad alpha with the left, and less consistently with the right hand (Table 3). On Table 3, although statistical significance is not always reached, most likely due to the current blocked experimental-design having a simple sample per participant, the mean amplitudes, *t*-values and their respective effect sizes tend to be greater for the left hand, except for the last second.

Overall, these observations suggest that immediately after contractions, an inhibitory reaction was triggered initially at the motor cortex, with greater intensity after left contractions, as indicated by increases in upper alpha/Mu amplitudes. Subsequently, this inhibition covered broader areas as shown by increases in the broad alpha amplitude in occipital, motor and frontal electrodes in the last three seconds. This inhibition was more consistent after left than after right contractions.

## Discussion

Numerous studies report that unilateral hand contractions produce particular behavioral effects after their termination [1–7], but the underlying mechanism is still not clear. One group of researchers [e.g., 4, 6, 7] proposed that unilateral hand contractions induce a shift in activation asymmetry to the contralateral brain hemisphere, which persists even after the termination of hand contractions. An alternative explanation, which comes from TMS research [9–11], states that a generalized state of reduced cortical activity emerges after the termination of unilateral hand contractions. The aim of the present experiment was to shed more light onto this issue by testing cortical activity during and after hand contractions. In particular, using the EEG alpha band, we contrasted if the hemispheric activation during contractions would prevail after their termination (the persistent activity model), or if a reduction of cortical activity would emerge (the reduced activity model). Our results favor the reduced activity model. Whereas a bilateral activation over motor areas was observed during contractions with any hand, we found a long lasting reduction of cortical activity below baseline over the whole scalp after contractions, which was especially evident when the left hand was contracted. These results suggest that the behavioral aftereffects induced through unilateral hand contractions are mediated by reduced cortical activity.

To the best of our knowledge, we are the first who compared the effects of unilateral hand contractions *after* their termination with a baseline before contractions using EEG. Increases in the alpha range, which reflect decreased cortical activity, have been related to reductions of the cortical response to TMS pulses [23–28]. Therefore, the currently observed increase of alpha after hand contractions (especially of the left hand) is similar to previous observations with TMS [e.g. 9, 10, 11]. Zanette et al. [10] observed a response reduction on the right hand for 35 minutes after one minute of repetitive abduction of the right thumb. Bäumer et al. [11] observed reduced cortical response to TMS for both hands, up to 15 minutes after executing pinch grips with only the left hand.

The reduction of cortical activity after hand contractions may be explained by inhibitory mechanisms. According to TMS scholars e.g., [10, 49], inhibitory mechanisms are massively activated after repetitive hand movements in order to control for neuronal hyper-excitability, and they are gradually turned off during a recovery period of several minutes. In our data, the onset of these inhibitory mechanisms was reflected by an enhancement in the upper alpha range over motor electrodes (known as “Mu rhythm” [43, 44; 48]) in the first second after the end of contractions, coinciding also with previous event-related studies of single hand movements [43, 44]. This rebound was bilateral after left hand contractions, and was greater than that after right contractions, which was only contralateral and did not reach statistical significance. The last three seconds of the period after contractions, showed enhancements of the broad alpha band that cover the initial upper alpha rebound, and mimic the broad alpha increase seen for the average of the whole two minute period. Moreover, the last three seconds also showed alpha enhancements in occipital and frontal areas, similar to the whole-scalp alpha enhancement seen for the two-minute average, which suggests inhibitory action also at these locations.

While TMS studies can prove a long lasting depression of cortical excitability over the involved sensory-motor cortex and its homologue contralateral region after repetitive movements, our current alpha band measurement revealed this reduction of activity also in regions away from sensory-motor areas. Distant regions uniformly decreased their activation levels for a long period after contractions. This might be accounted for by the complex interactions that occur over the cortex during hand movements. Inhibition of occipital areas during single hand movements has previously been reported [47]. In this experiment, occipital electrodes were the only ones to increase, albeit slightly, their alpha amplitudes during contractions. As already shown, this inhibition carried on and intensified towards the post-contractions period. On the other side, frontal areas are involved in the control of hand movements [43, 50, 51] and, like motor areas, need to be down-regulated after movement implementation [43, 51]. During contractions, we observed significant anterior activations at FC3, FC4, Fp1, and F3, which became subsequently inhibited along the period after contractions. In a study with epileptic patients, Derambure et al [51] observed that patients with focal motor seizures, as well as those with temporal lobe epilepsy took considerably longer to deactivate sensory-motor and frontal regions after single hand movements compared to healthy participants, further supporting the importance of these inhibitory mechanisms in healthy brains.

We assume that during contractions, the activation of the network involved in motor action further influenced other cortical regions through complex axonal pathways [52, 53], but local inhibitory mechanisms prevented their over-excitation [10, 41, 49, 52, 54 **].** Inhibitory mechanisms acting at separate locations from active cortex have been proposed [41, 52]. Although these inhibitory mechanisms become most evident in studies of epilepsy, they are presumably analogous to those present in healthy population [41, 52, 54–56]. In epileptic patients, the fact that pathological activity can quickly spread to distant, even multi-lobar regions supports that long axonal pathways can transfer activity beyond the trigger area [52, 53]. Furthermore, when seizures fail to spread, activity at surrounding and distant regions is still influenced by the ictal focus, before it normalizes through the intervention of local inhibitory mechanisms [52]. In support of the former arguments, it has been reported that clenching the fist can produce generalized spike-wave discharges in epileptic patients [57]. The aforementioned inhibitory restrain has also been observed *in vitro* [52, 55, 56] and is presumed to be the analogous to the surround inhibition seen during normal motor performance [41, 47, 58]. In our sample with healthy population, we propose that this traveling activity from the motor network is balanced out by local inhibitory mechanisms without reaching pathological levels. As mentioned by Trevelyan et al. [55], the same inhibitory mechanisms may restrain less intense forms of activity.

Altogether, we propose that the observed increment in alpha amplitudes after contractions results from inhibitory mechanisms that first activate during contractions to control cortical excitability [10, 41, 49], prevent the spread of activity [10, 41, 49, 52, 54], and provide movement specificity [47, 58]. The long lasting alpha increase parallels the recovery period seen in TMS experiments [9–11], and may then reflect a longer lasting action of the inhibitory mechanisms after hand contractions are finished, as proposed by Zanette et al. [10] and Bonato et al. [49]. Notably, the strength of cortical inhibition after unilateral hand contractions was observed at all regions after left-hand contractions while for the right hand, this effect was much weaker, and seldom reached statistical significance. We propose this may result from the greater level of white matter and connectivity of the right hemisphere (which mostly commands the left hand) towards the rest of the brain [59–63], prompting inhibitory mechanisms to a greater extent.

Cortical activity *during* hand contractions also deserves mention. Contrary to the persistent activity model, we found no effect over hemispheric activation asymmetries during hand contractions (neither after contractions). Thus, our results are at odds with previous EEG studies [1, 2, 5]. This might be due to differences in experimental design. The previous experiments had been between-subjects, whereas we used a within-subject design. Similar studies with within-subjects designs also observed bilateral sensorimotor activations during unilateral hand contractions. For example, using functional magnetic resonance imaging (fMRI), Liu et al. [64] observed steady increase of fMRI signal in both contralateral and ipsilateral hemispheres during hand contractions. This observation also coincides with other EEG studies which used different kinds of repetitive unilateral movements like finger taping [65] or rhythmic flexion movements of the index finger [50]. Using positron emission tomography (PET) and TMS, Dettmers et al. [66] observed an increase in regional blood flow and of cortical excitability (both implying cortical activation) of both motor cortices during unilateral finger tapping. It is thus possible that the asymmetry effects become visible especially when comparing large polls of subjects in the left against subjects in the right hand contraction conditions.

Alpha asymmetries between the two brain hemispheres were also not systematically modified after hand contractions, meaning that regardless of general changes in activity levels at each phase, relative activity between homolog locations at each side remained constant. This observation is especially relevant regarding anterior brain asymmetries, which are known to influence mood and emotional predispositions [67– 69]. While left anterior asymmetries at rest (smaller left alpha amplitudes) are associated with tendencies towards parasympathetic activation, approach behavior, and positive affect [67–69], right sided asymmetries are related with sympathetic activation, avoidance behavior, and negative affect [67–69]. In EEG emotion research, anterior asymmetries are most commonly assessed between electrodes F3 on the left and F4 on the right [68–70], which tap on activity of the dorsolateral pre-frontal cortex as part of a broader emotional regulation system [68, 70, 71]. In our sample, these electrode pairs consistently showed greater left sided activity (smaller left alpha amplitudes, Fig 1). Other electrode pairs used in emotion research such as FC3-FC4 [68], and C3-C4 [67, 72] also kept constant left sided asymmetry throughout our experiment. These constant asymmetry levels suggest that left hand contractions might be safely used to enhance alpha amplitudes without the risk of inducing a potentially unwanted right-sided frontal asymmetry.

In sum, our results suggest that unilateral hand contractions result in a state of reduced cortical activity (and not the persistent activation asymmetry) after their termination, which is more pronounced if the left hand was used. This seems to be a plausible mechanism underlying the subsequent behavioral effects reported in previous experiments [1–7]. Consistent with this, prior laboratory research reports that elevated alpha levels at rest (indicating cortical inhibition) improve the brain’s engagement in subsequent information processing [12–15]. Research in sport and rehabilitation performance further supports that global reductions in basal cortical activity facilitate task-specific activations and reduce competition from non-essential cortical regions [16–21, 73]. The present experiment included no tasks following hand contractions in order to prevent task-related activations to affect the measurement. Future studies should attempt to include a test of the mediating effect of induced alpha enhancement on subsequent behavior.

## Conclusions

The current experiment tested the state of cortical activity during and after unilateral hand contractions. The EEG reported alpha band assessment revealed that hand contractions produce bilateral activations of the motor cortex during their execution. But after contractions, a state of globally reduced cortical activity emerges, especially when the left hand was used. This state is most likely produced by inhibitory mechanisms activated during repetitive contractions, which outlast their termination. It may be proposed that once this state is induced, it facilitates performance in subsequent tasks by facilitating task-specific cortical activations and preventing interference from other, nonessential cortical regions. In addition, the increase of amplitudes in the alpha range through hand contractions is similar to that induced through other techniques like repetitive transcranial magnetic stimulation [29] or transcranial alternating current stimulation [22, 30]. Therefore, unilateral hand contractions, especially with the left hand, also show promissory possibilities for their use in clinical settings.

## Supporting Information

S1 Annex Differences between visual and sensorimotor regions during contractions. (DOCX)

S2 Annex Alternative Electrode Referencing. (DOCX)

S1 Fig Comparison of alpha amplitudes before and after contractions of each hand for each subgroup. A) Left-first subgroup, left contractions. B) Left-first subgroup, right contractions. C) Right-first subgroup, left contractions. D) Right-first subgroup, right contractions.
(TIF)

S2 Fig Highlight of occipital effects observed during contractions. A) For the left hand-block. B) For the right hand-block. The plot is taken from Fig 1 with highlights for occipital electrodes. The accompanying difference maps are obtained by subtracting the phase during contractions from the baseline. The scale has been adjusted to illustrate occipital effects.
(TIF)

S3 Fig Average Reference plot of the alpha amplitudes at each electrode before, during and after contractions. A) For the left hand-block. B) For the right hand-block. Accompanying difference maps indicate the distribution of amplitude changes on the scalp when subtracting the baseline before contractions from the phases during and after contractions.
(TIF)

S4 Fig Surface Laplacian plot of the alpha amplitudes at each electrode before, during and after contractions. A) For the left hand-block. B) For the right hand-block. Accompanying difference maps indicate the distribution of amplitude changes on the scalp when subtracting the baseline before contractions from the phases during and after contractions.
(TIF)

S1 Table Mean (*SD*) alpha amplitudes at each electrode before left and right contractions with *t*-scores and effect sizes for differences between both baselines. (DOCX)

S2 Table *t*-scores and effect sizes for differences in alpha amplitudes between the phases before and after hand contractions for each electrode and each hand according to which hand-block was performed first. (DOCX)
