# Correction: Unilateral Left-Hand Contractions Produce Widespread Depression of Cortical Activity after Their Execution

**DOI:** 10.1371/journal.pone.0150048

**Published:** 2016-02-19

**Authors:** Fernando Cross-Villasana, Peter Gröpel, Michael Doppelmayr, Jürgen Beckmann

The legend of Fig 2 appears incorrectly in the published article. Please see the correct Fig 2 and its legend below.

**Fig 2:**
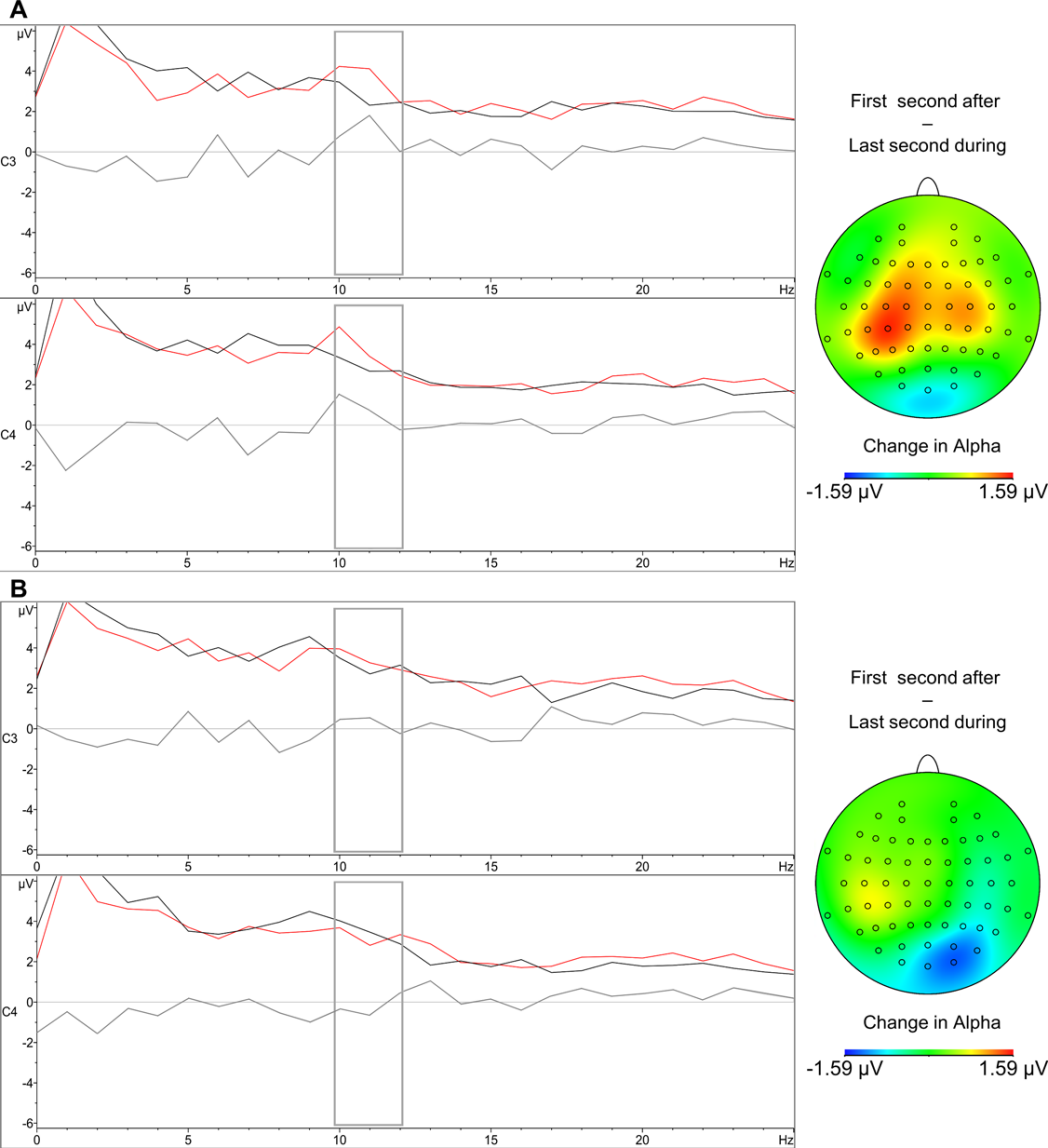
Spectral plot of the last second during contractions (black), first second after contractions (red), and their difference (gray). A) For the left hand-block. B) For the right hand-block. The gray rectangle highlights the upper alpha band. Accompanying difference maps illustrate the difference over the scalp when subtracting the last second of contractions, from the first second after contractions.
