# Supplementary material for: Unilateral Left-Hand Contractions Produce Widespread Depression of Cortical Activity after Their Execution
Source: PLoS One. 2015 Dec 28;10(12):e0145867. doi: 10.1371/journal.pone.0145867 (PMC4692494; doi:10.1371/journal.pone.0145867)
Supplement: S1 Annex — (DOCX) [file pone.0145867.s001.docx]

**S1 ANNEX. Differences between visual and sensorimotor regions during contractions.**

An interaction between electrode and phase indicated that differences among electrodes varied at each phase. In part, this owes to the fact that electrodes C3, C4, CP3, and CP4, which reflect sensorimotor areas, showed the greatest bilateral decrease in alpha amplitudes during contractions (see Table 1 and Table 2 in the main text). On the other side, electrodes O1 and O2, reflecting visual areas, slightly increased their amplitudes during contractions, except for O2 for the right hand, which slightly decreased (Table 1 in the main text). Such a contrast between central and occipital electrodes enhanced their differences during contractions. To better visualize this contrast, Figure S2 displays the same difference maps of Figure 1 with an adjusted scale to enhance the slight increase of alpha amplitude in occipital regions.

For post-hoc analysis, the differences between electrodes O1 and O2 with each of the other electrodes were calculated at each phase for each hand. Afterwards, the obtained differences at each phase were compared with each other using *t*-tests. For the left hand, Table A in this supplement shows the obtained difference values at each phase and Table B shows the *t*-values obtained when those differences are compared to each other. For the right hand, Table C displays the differences, and Table D the results of the comparisons between those differences.

As can be observed in Table B for the left hand and Table D for the right hand, the difference between occipital electrodes and central electrodes during contractions is consistently greater than that before contractions, as well as after contractions. Further, when comparing the difference among these electrodes after contractions with that before contractions, this discrepancy disappears. The described pattern was observed with the greatest consistency only between occipital and central electrodes for both hands. With occipital vs parietal electrodes the pattern was less consistent, and with other electrodes is not observed.

This observation is in line with the findings reported by Pfurtscheller and Lopes da Silva [47], who used event-related synchronization/desynchronization (ERS/ERD) of the alpha band to observe a simultaneous activation of motor areas and deactivation of the visual areas over the scalp during hand movements.

**Table A**

|  | Mean Differences | | | | | |
| --- | --- | --- | --- | --- | --- | --- |
|  | O1 | | | O2 | | |
|  | Before | During | After | Before | During | After |
| C3 | .45 (.45) | .73 (.77) | .51 (.59) | .42 (.52) | .70 (.77) | .50 (.62) |
| C4 | .41 (.37) | .68 (.66) | .47 (.55) | .38 (.44) | .64 (.67) | .46 (.59) |
| CP3 | .29 (.41) | .62 (.73) | .34 (.53) | .26 (.50) | .59 (.74) | .32 (.61) |
| CP4 | .24 (.31) | .57 (.60) | .29 (.45) | .21 (.39) | .54 (.61) | .28 (.51) |
|  |  |  |  |  |  |  |
| Fp1 | .73 (.44) | .92 (.68) | .83 (.66) | .70 (.49) | .88 (.68) | .82 (.66) |
| Fp2 | .76 (.44) | .93 (.71) | .88 (.66) | .72 (.48) | .90 (.70) | .87 (.65) |
| F3 | .60 (.40) | .77 (.69) | .67 (.56) | .56 (.46) | .74 (.69) | .65 (.59) |
| F4 | .57 (.42) | .74 (.72) | .66 (.58) | .54 (.49) | .70 (.72) | .65 (.61) |
| F7 | .69 (.42) | .83 (.68) | .75 (.51) | .66 (.47) | .80 (.68) | .74 (.55) |
| F8 | .69 (.45) | .85 (.73) | .82 (.65) | .66 (.52) | .82 (.73) | .81 (.68) |
| FC3 | .57 (.41) | .78 (.79) | .63 (.57) | .54 (.49) | .74 (.78) | .62 (.61) |
| FC4 | .55 (.40) | .70 (.70) | .62 (.57) | .51 (.47) | .66 (.70) | .61 (.60) |
| FT7 | .74 (.41) | .89 (.77) | .79 (.58) | .71 (.47) | .86 (.77) | .78 (.63) |
| FT8 | .81 (.51) | .91 (.81) | .91 (.70) | .78 (.57) | .87 (.83) | .90 (.74) |
| T7 | .90 (.43) | 1.05 (.77) | .95 (.52) | .87 (.48) | 1.01 (.78) | .94 (.57) |
| T8 | .93 (.60) | 1.01 (.78) | .99 (.70) | .90 (.67) | .98 (.80) | .97 (.78) |
| P7 | .27 (.30) | .51 (.52) | .32 (.37) | .24 (.39) | .47 (.54) | .31 (.53) |
| P8 | .30 (.39) | .45 (.44) | .29 (.40) | .27 (.46) | .42 (.47) | .28 (.48) |
| P3 | .14 (.29) | .40 (.48) | .18 (.40) | .11 (.37) | .36 (.49) | .16 (.50) |
| P4 | .16 (.24) | .36 (.40) | .17 (.39) | .13 (.32) | .32 (.39) | .16 (.42) |

Mean (SD) of the differences in alpha amplitudes between electrodes O1 and O2 and the rest of the electrodes across each measuring phase for the left hand-block.

**Table B**

|  | *t*-scores | | | | | |
| --- | --- | --- | --- | --- | --- | --- |
|  | O1 | | | O2 | | |
|  | Before vs During | During vs After | Before vs  After | Before vs During | During vs After | Before vs  After |
| C3 | -2.25* | 2.21* | -1.00 | -2.51* | 2.06 | -1.84 |
| C4 | -2.30* | 2.39* | -.66 | -2.61* | 2.13* | -1.18 |
| CP3 | -2.71* | 2.82* | -.68 | -2.97* | 2.42* | -1.21 |
| CP4 | -2.47* | 3.01* | -.52 | -2.70* | 2.57* | -.94 |
|  |  |  |  |  |  |  |
| Fp1 | -1.73 | 0.94 | -1.25 | -2.02 | 0.76 | -2.13* |
| Fp2 | -1.69 | 0.62 | -1.61 | -2.00 | 0.39 | -2.67* |
| F3 | -1.60 | 1.28 | -1.01 | -1.83 | 1.05 | -1.86 |
| F4 | -1.59 | 0.98 | -1.44 | -1.82 | 0.70 | - 2.54 |
| F7 | -1.28 | 0.92 | -1.06 | -1.48 | 0.73 | -2.37* |
| F8 | -1.56 | 0.41 | -1.69 | -1.84 | 0.09 | -2.61 |
| FC3 | -1.73 | 1.80 | -0.98 | -1.95 | 1.49 | -1.80 |
| FC4 | -1.54 | 1.02 | -1.09 | -1.77 | 0.74 | -2.02 |
| FT7 | -1.28 | 1.31 | -0.71 | -1.43 | 1.05 | -1.27 |
| FT8 | -0.91 | -0.05 | -1.27 | -1.01 | -0.36 | -1.98 |
| T7 | -1.05 | 0.94 | -0.57 | -1.15 | 0.74 | -1.10 |
| T8 | -0.72 | 0.39 | -0.63 | -0.81 | 0.08 | -1.11 |
| P7 | -2.39* | 2.51* | -.87 | -2.72* | 1.80 | -1.30 |
| P8 | -2.48* | 2.76* | .21 | -3.13* | 2.52* | -.241 |
| P3 | -2.58* | 2.70* | -.60 | -2.79* | 2.03 | -1.01 |
| P4 | -2.39* | 2.82* | -.21 | -2.73* | 1.76 | -.68 |

*t*-scores for differences in the disparity of electrodes O1 and O2 against other electrodes during the recording phases compared to each other during the left hand-block.

*indicates significance *p* < .05 (uncorrected), and **indicates significance *p* < .001 (uncorrected).

**Table C**

|  | Mean Differences | | | | | |
| --- | --- | --- | --- | --- | --- | --- |
|  | O1 | | | O2 | | |
|  | Before | During | After | Before | During | After |
| C3 | .51 (.58) | .78 (.82) | .57 (.66) | .47 (56) | .80 (.79) | .55 (.67) |
| C4 | .43 (.52) | .71 (.72) | .48 (.50) | .40 (.54) | .72 (.72) | .47 (.55) |
| CP3 | .36 (.55) | .68 (.82) | .41 (.62) | .32 (.56) | .70 (.80) | .40 (.64) |
| CP4 | .29 (.45) | .55 (.64) | .28 (.44) | .25 (.47) | .57 (.64) | .27 (.49) |
|  |  |  |  |  |  |  |
| Fp1 | .75 (.53) | .88 (.68) | .83 (.53) | .71 (.55) | .90 (.69) | .81 (.60) |
| Fp2 | .78 (.53) | .92 (.71) | .89 (.55) | .74 (.54) | .93 (.72) | .87 (.60) |
| F3 | .63 (.49) | .77 (.67) | .69 (.50) | .60 (.52) | .78 (.66) | .68 (.57) |
| F4 | .59 (.54) | .73 (.72) | .67 (.56) | .55 (.57) | .74 (.74) | .66 (.64) |
| F7 | .72 (.48) | .82 (.64) | .76 (.46) | .69 (.51) | .83 (.65) | .75 (.54) |
| F8 | .75 (.58) | .84 (.72) | .82 (.60) | .71 (.59) | .85 (.74) | .81 (.66) |
| FC3 | .61 (.53) | .79 (.75) | .68 (.58) | .57 (.54) | .80 (.73) | .67 (.62) |
| FC4 | .58 (.56) | .75 (.77) | .67 (.60) | .54 (.57) | .76 (.76) | .66 (.64) |
| FT7 | .82 (.54) | .90 (.77) | .81 (.55) | .78 (.55) | .91 (.76) | .79 (.59) |
| FT8 | .87 (.66) | .96 (.81) | .94 (.66) | .84 (.66) | .97 (.83) | .92 (.71) |
| T7 | .94 (.52) | 1.04 (.81) | .92 (.58) | .90 (.52) | 1.05 (.79) | .91 (.61) |
| T8 | 1.00 (.65) | 1.08 (.80) | .96 (.66) | .96 (.67) | 1.09 (.82) | .95 (.72) |
| P7 | .33 (.34) | .52 (.50) | .33 (.35) | .29 (.41) | .53 (.53) | .32 (.46) |
| P8 | .31 (.42) | .41 (.44) | .26 (.45) | .28 (.44) | .42 (.46) | .24 (.50) |
| P3 | .19 (.44) | .44 (.62) | .22 (.42) | .15 (.46) | .45 (.60) | .20 (.46) |
| P4 | .20 (.41) | .32 (.45) | .18 (.41) | .16 (.40) | .33 (.43) | .17 (.43) |

Mean (SD) of the differences in alpha amplitudes between electrodes O1 and O2 and the rest of the electrodes across each measuring phase for the right hand-block.

**Table D**

|  | *t*-scores | | | | | |
| --- | --- | --- | --- | --- | --- | --- |
|  | O1 | | | O2 | | |
|  | Before vs During | During vs After | Before vs  After | Before vs During | During vs After | Before vs  After |
| C3 | -2.58* | 2.11* | -1.15 | -3.03* | 2.36* | -1.58 |
| C4 | -2.90* | 2.16* | -1.02 | -3.03* | 2.44* | -1.51 |
| CP3 | -2.73* | 2.50* | -.89 | -3.03* | 2.79 | -1.22 |
| CP4 | -2.59* | 2.30* | .09 | -2.88* | 2.69* | -.472 |
|  |  |  |  |  |  |  |
| Fp1 | -1.80 | 0.83 | -3.17* | -2.18* | 1.12 | -3.12* |
| Fp2 | -1.90 | 0.46 | -4.24** | -2.30* | 0.78 | -4.07** |
| F3 | -1.65 | 0.99 | -1.57 | -2.07 | 1.27 | -1.93 |
| F4 | -2.02 | 0.87 | -2.54* | -2.34* | 1.12 | -3.14* |
| F7 | -1.31 | 0.79 | -1.17 | -1.80 | 1.10 | -1.64 |
| F8 | -1.56 | 0.33 | -2.32* | -1.93 | 0.66 | -2.66* |
| FC3 | -2.25* | 1.29 | -1.78 | -2.74* | 1.61 | -2.14* |
| FC4 | -2.30* | 1.04 | -2.96* | -2.58* | 1.30 | -3.38* |
| FT7 | -1.05 | 1.02 | 0.19 | -1.56 | 1.31 | -0.21 |
| FT8 | -1.20 | 0.26 | -1.26 | -1.59 | 0.56 | -1.68 |
| T7 | -0.88 | 0.92 | 0.19 | -1.31 | 1.11 | -0.20 |
| T8 | -1.09 | 1.22 | 0.59 | -1.53 | 1.39 | 0.20 |
| P7 | -2.39* | 2.08 | -.08 | -2.51* | 2.12* | -.585 |
| P8 | -1.70 | 2.29* | 1.67 | -2.73* | 3.08* | .95 |
| P3 | -2.38* | 2.22* | -.729 | -2.67* | 2.52* | -1.14 |
| P4 | -1.55 | 1.32 | .479 | -2.43* | 1.71 | -.29 |

*t*-scores for differences in the disparity of electrodes O1 and O2 against other electrodes during the recording phases compared to each other during the right hand-block.

*indicates significance *p* < .05 (uncorrected), and **indicates significance *p* < .001 (uncorrected).
