# Supplementary material for: Unilateral Left-Hand Contractions Produce Widespread Depression of Cortical Activity after Their Execution
Source: PLoS One. 2015 Dec 28;10(12):e0145867. doi: 10.1371/journal.pone.0145867 (PMC4692494; doi:10.1371/journal.pone.0145867)
Supplement: S2 Annex — (DOCX) [file pone.0145867.s002.docx]

**S2 ANNEX. Alternative Electrode Referencing**

In order to corroborate the results obtained with the linked mastoids reference, the data was further analyzed using an average-reference scheme, and a reference-free surface Laplacian approach.

**Average Reference**

The average reference procedure showed results that largely mirrored those obtained with a linked-mastoids reference, with main effects of phase *F*(2, 38) = 22.88, *p* = .001, $\eta_{p}^{2}$ = .55 and electrode *F*(21, 399) = 28.96, *p* = .001, $\eta_{p}^{2}$ = .60; no significant effect for hand-block *F*(1, 19) = .598, *p* = .45, $\eta_{p}^{2}$ = .03; an interaction between electrode and phase, *F*(42, 798) = 8.23, *p* = .001, $\eta_{p}^{2}$ = .30; and no significant three way interaction between all factors *F*(42, 798) = .68, *p* = .937, $\eta_{p}^{2}$ = .04.

The interaction between phase and hand-block however, did not reach statistical significance *F*(2, 38) = 2.43, *p* = .101, $\eta_{p}^{2}$ = .11. Post-hoc *t*-tests comparing the value of each electrode at each phase (Table A in this supplement) showed that although alpha amplitudes increased after left-hand contractions compared to baseline (S3 Figure), fewer electrodes reached statistical significance, or they did to a lesser degree.

As with the linked-mastoids reference, a repeated measures ANOVA with the asymmetry ratios showed no main effects and there was no significant interaction between phase and hand-block *F*(2, 38) = .21, *p* = .814, $\eta_{p}^{2}$ = .01, confirming that alpha asymmetry ratios were not affected my hand contractions.

**Table A.**

| **Electrode Position** | **Left Hand Contractions** | | | | **Right Hand Contractions** | | | |
| --- | --- | --- | --- | --- | --- | --- | --- | --- |
|  | **Before vs. During** | | **Before vs. After** | | **Before vs. During** | | **Before vs. After** | |
|  | ***t*(19)** | ***d_z_*** | ***t*(19)** | ***d_z_*** | ***t*(19)** | ***d_z_*** | ***t*(19)** | ***d_z_*** |
| Fp1 | 3.03* | .68 | -2.92* | .65 | 2.49 | .56 | -.41 | .09 |
| Fp2 | 2.94* | .66 | -2.17 | .49 | 2.96* | .66 | .34 | .08 |
| F3 | 3.78** | .85 | -3.51** | .78 | 2.61 | .58 | -.59 | .13 |
| F4 | 2.99* | .67 | -2.38 | .53 | 3.04* | .68 | -.03 | .01 |
| F7 | 2.33 | .52 | -3.00* | .67 | 2.01 | .45 | -1.11 | .25 |
| F8 | 2.18 | .49 | -1.47 | .33 | 2.17 | .49 | -.80 | .18 |
| C3 | 4.44** | .99 | -2.46 | .55 | 3.92** | .88 | -.18 | .04 |
| C4 | 4.10** | .92 | -2.59* | .58 | 4.25** | .95 | -.77 | .17 |
| FC3 | 3.16* | .71 | -3.09* | .69 | 3.17** | .71 | -.12 | .03 |
| FC4 | 2.91* | .65 | -2.49 | .56 | 3.23** | .72 | .43 | .10 |
| FT7 | 2.17 | .49 | -3.69** | .83 | 2.21 | .49 | -1.47 | .33 |
| FT8 | 1.98 | .44 | -2.95* | .66 | 2.04 | .46 | -1.37 | .31 |
| CP3 | 5.31** | 1.19 | -2.28 | .51 | 4.50** | 1.01 | .01 | .00 |
| CP4 | 4.36** | .97 | -3.40** | .76 | 5.07** | 1.13 | -1.77 | .40 |
| T7 | 2.39 | .53 | -3.60** | .80 | 2.29 | .51 | -1.18 | .26 |
| T8 | 1.59 | .36 | -4.11** | .92 | 2.08 | .47 | -3.27** | .73 |
| P7 | 3.16* | .71 | -3.43** | .77 | 2.82* | .63 | -1.00 | .22 |
| P8 | 1.18 | .26 | -4.06** | .91 | 1.21 | .27 | -4.92** | 1.10 |
| P3 | 3.72** | .83 | -2.75* | .61 | 4.10** | .92 | -.23 | .05 |
| P4 | 2.40 | .54 | -4.16** | .93 | 2.99* | .67 | -1.96 | .44 |
| O1 | -0.27 | .06 | -2.30 | .51 | 0.14 | .03 | -1.02 | .23 |
| O2 | -0.46 | .10 | -3.18** | .71 | -0.45 | .10 | -2.38 | .53 |

*t*-scores and effect sizes for differences in alpha amplitudes between the phases before and during and the phases before and after hand contractions for each electrode and each hand using Average Reference.

*indicates significance *p* < .016, and **indicates significance *p* < .003 (corrected for multiple (3) comparisons – Bonferroni).

**Surface Laplacian**

A surface Laplacian [38] was further implemented. Given the observed widespread increase of alpha amplitudes after left contractions with the linked mastoids and average references, a spline order *m* = 7 and smoothing constant λ = 0.00001 were used for the surface Laplacian [39]. These parameters, should prevent high-pass spatial filtering by the surface Laplacian and hence emphasize global over local features of the EEG [39-42] such as the current widespread increase of alpha amplitudes.

The results that coincided with the linked-mastoid reference, showing main effects of phase *F*(2, 38) = 18.84, *p* = .001, $\eta_{p}^{2}$ = .50 and electrode *F*(21, 399) = 31.36, *p* = .001, $\eta_{p}^{2}$ = .62; no significant effect for hand-block *F*(1, 19) = 1.08, *p* = .311, $\eta_{p}^{2}$ = .05; an interaction between electrode and phase, *F*(42, 798) = 7.24, *p* = .001, $\eta_{p}^{2}$ = .28; and no significant three way interaction *F*(42, 798) = .430, *p* = .999, $\eta_{p}^{2}$ = .02.

Most importantly, the interaction between phase and hand-block was significant *F*(2, 38) = 3.29, *p* = .048, $\eta_{p}^{2}$ = .15, as with linked-mastoids. Post-hoc *t*-tests in Table B of this supplement show that while all electrodes except for CP3 show a significant increase in alpha amplitudes after left hand contractions (S4 Figure), only electrode P8 does so after right hand contractions.

As with the linked-mastoids reference, a repeated measures ANOVA with the asymmetry ratios showed no main effects and there was no significant interaction between phase and hand-block *F*(2, 38) = .76, *p* = .468, $\eta_{p}^{2}$ = .04, confirming that alpha asymmetry ratios were not affected my hand contractions.

**Table B.**

| **Electrode Position** | **Left Hand Contractions** | | | | **Right Hand Contractions** | | | |
| --- | --- | --- | --- | --- | --- | --- | --- | --- |
|  | **Before vs. During** | | **Before vs. After** | | **Before vs. During** | | **Before vs. After** | |
|  | ***t*(19)** | ***d_z_*** | ***t*(19)** | ***d_z_*** | ***t*(19)** | ***d_z_*** | ***t*(19)** | ***d_z_*** |
| Fp1 | 5.12** | 1.14 | -3.71** | .83 | 4.94** | 1.10 | -.33 | .07 |
| Fp2 | 4.18** | .93 | -4.64** | 1.04 | 4.59** | 1.03 | -1.53 | .34 |
| F3 | 4.34** | .97 | -3.34** | .75 | 4.27** | .95 | .05 | .01 |
| F4 | 2.40 | .54 | -3.93** | .88 | 3.23** | .72 | -1.09 | .24 |
| F7 | 0.97 | .22 | -3.08* | .69 | 1.73 | .39 | .34 | .08 |
| F8 | -0.10 | .02 | -3.64** | .81 | 0.39 | .09 | -1.41 | .32 |
| C3 | 2.96* | .66 | -2.94* | .66 | 3.11* | .70 | -.87 | .19 |
| C4 | 2.50 | .56 | -2.95* | .66 | 2.99* | .67 | -1.11 | .25 |
| FC3 | 2.37 | .53 | -3.08* | .69 | 2.49 | .56 | -.41 | .09 |
| FC4 | 1.42 | .32 | -3.30** | .74 | 2.11 | .47 | -.89 | .20 |
| FT7 | 1.07 | .24 | -3.25** | .73 | 1.33 | .30 | -.48 | .11 |
| FT8 | 0.30 | .07 | -4.28** | .96 | 0.71 | .16 | -1.83 | .41 |
| CP3 | 4.48** | 1.00 | -2.63* | .59 | 4.43** | .99 | -.70 | .16 |
| CP4 | 3.37** | .75 | -3.21** | .72 | 4.20** | .94 | -1.30 | .29 |
| T7 | 2.86* | .64 | -3.51** | .78 | 2.64* | .59 | -1.05 | .23 |
| T8 | 2.27 | .51 | -5.24** | 1.17 | 2.78* | .62 | -2.27 | .51 |
| P7 | 3.62** | .81 | -3.02* | .68 | 4.14** | .93 | -.51 | .11 |
| P8 | 2.69* | .60 | -4.59** | 1.03 | 3.54** | .79 | -2.80* | .63 |
| P3 | 4.01** | .90 | -2.70* | .60 | 4.10** | .92 | -.35 | .08 |
| P4 | 2.67* | .60 | -3.87** | .87 | 3.21** | .72 | -1.72 | .38 |
| O1 | 0.63 | .14 | -2.96* | .66 | 0.92 | .21 | -1.01 | .23 |
| O2 | 0.10 | .02 | -3.44** | .77 | 0.06 | .01 | -2.51 | .56 |

*t*-scores and effect sizes for differences in alpha amplitudes between the phases before and during and the phases before and after hand contractions for each electrode and each hand.

*indicates significance *p* < .016, and **indicates significance *p* < .003 (corrected for multiple (3) comparisons – Bonferroni).
