# Supplementary figures and images for: Unilateral Left-Hand Contractions Produce Widespread Depression of Cortical Activity after Their Execution
Source: PLoS One. 2015 Dec 28;10(12):e0145867. doi: 10.1371/journal.pone.0145867 (PMC4692494; doi:10.1371/journal.pone.0145867)

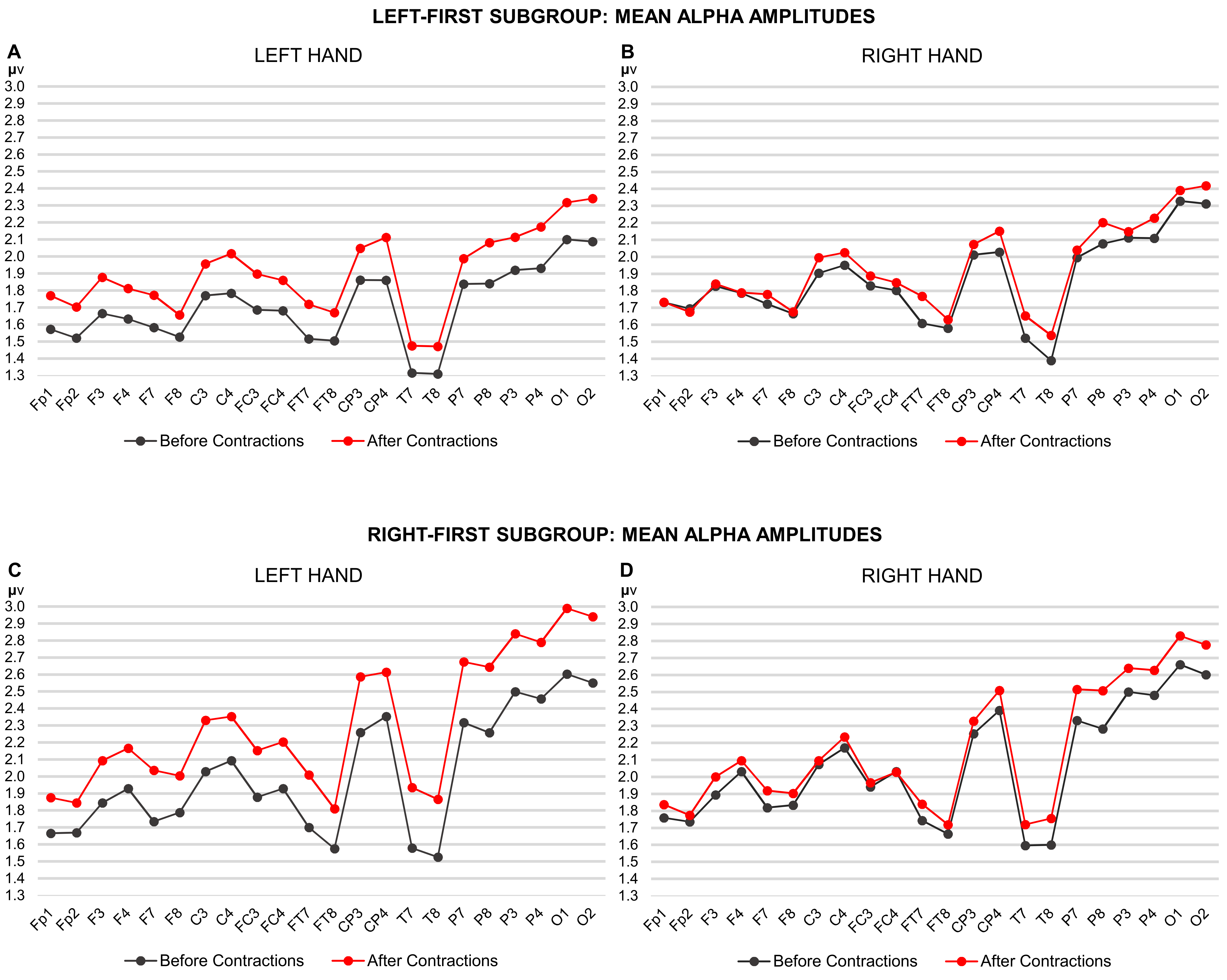

Supplement: S1 Fig — A) Left-first subgroup, left contractions. B) Left-first subgroup, right contractions. C) Right-first subgroup, left contractions. D) Right-first subgroup, right contractions. (TIF) [file pone.0145867.s003.tif]

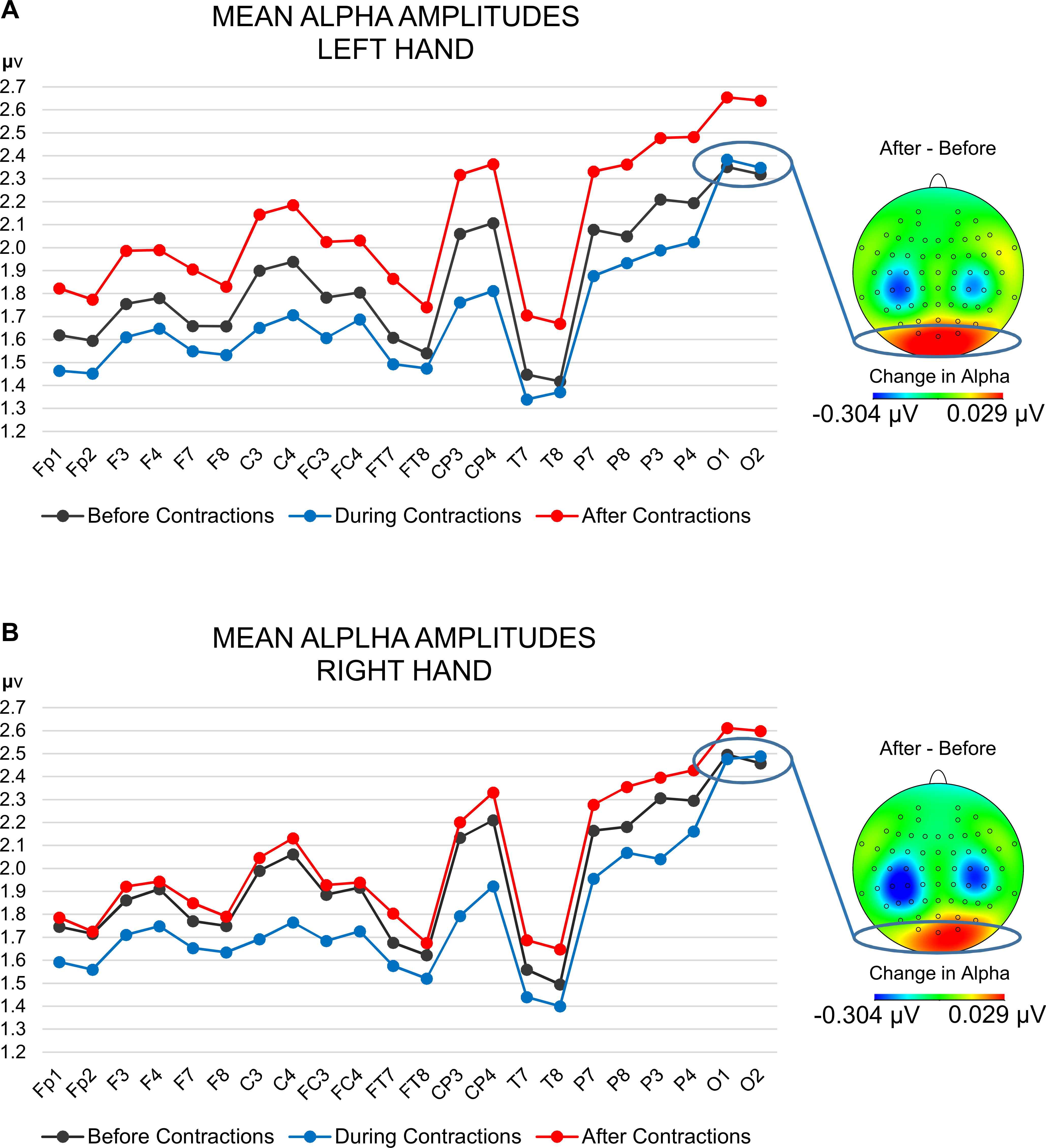

Supplement: S2 Fig — A) For the left hand-block. B) For the right hand-block. The plot is taken from Fig 1 with highlights for occipital electrodes. The accompanying difference maps are obtained by subtracting the phase during contractions from the baseline. The scale has been adjusted to illustrate occipital effects. (TIF) [file pone.0145867.s004.tif]

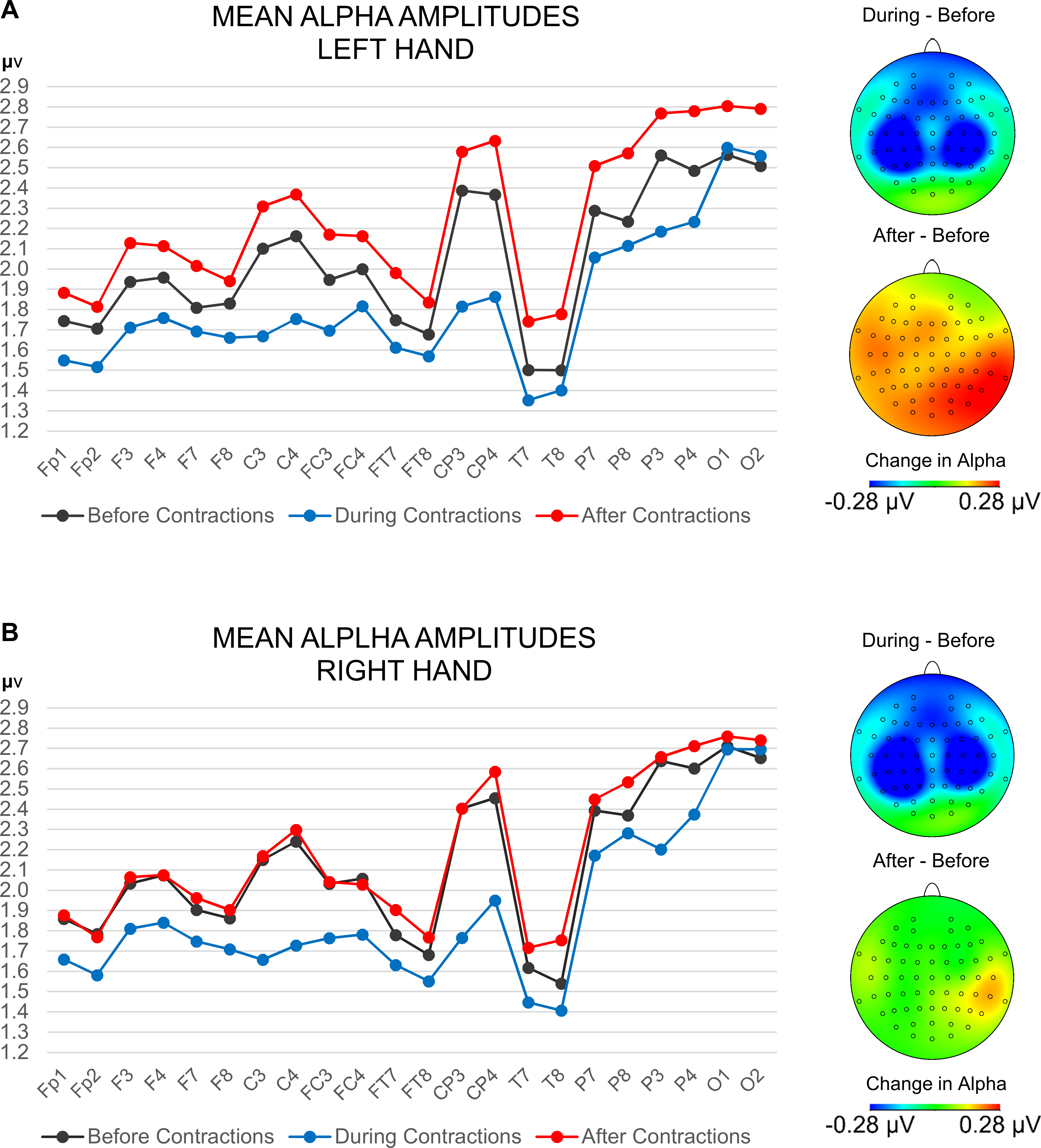

Supplement: S3 Fig — A) For the left hand-block. B) For the right hand-block. Accompanying difference maps indicate the distribution of amplitude changes on the scalp when subtracting the baseline before contractions from the phases during and after contractions. (TIF) [file pone.0145867.s005.tif]

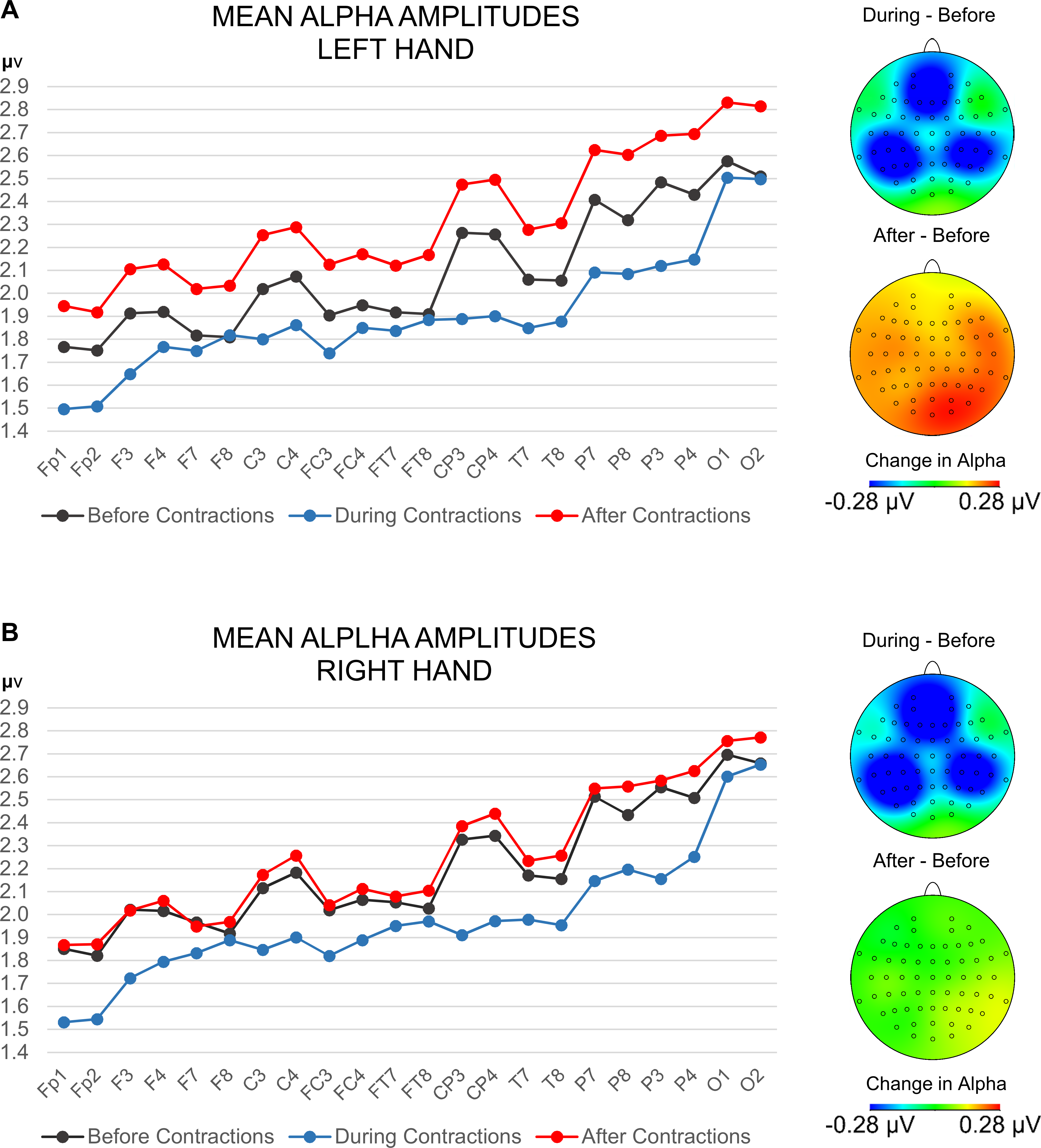

Supplement: S4 Fig — A) For the left hand-block. B) For the right hand-block. Accompanying difference maps indicate the distribution of amplitude changes on the scalp when subtracting the baseline before contractions from the phases during and after contractions. (TIF) [file pone.0145867.s006.tif]
