# Supplementary material for: Unilateral Left-Hand Contractions Produce Widespread Depression of Cortical Activity after Their Execution
Source: PLoS One. 2015 Dec 28;10(12):e0145867. doi: 10.1371/journal.pone.0145867 (PMC4692494; doi:10.1371/journal.pone.0145867)
Supplement: S1 Table — (DOCX) [file pone.0145867.s007.docx]

**S1 Table.** Mean (*SD*) alpha amplitudes at each electrode before left and right contractions with *t*-scores and effect sizes for differences between both baselines.

| **Electrode Position** | **Before Left**  **Contractions** | **Before Right**  **Contractions** | ***t*(19)** | ***d_z_*** |
| --- | --- | --- | --- | --- |
| Fp1 | 1.62 (.48) | 1.75 (.59) | -2.29* | .51 |
| Fp2 | 1.59 (.47) | 1.72 (.54) | -2.15* | .48 |
| F3 | 1.76 (.59) | 1.86 (.67) | -2.27* | .51 |
| F4 | 1.78 (.55) | 1.91 (.64) | -2.99** | .67 |
| F7 | 1.66 (.54) | 1.77 (.65) | -2.39* | .53 |
| F8 | 1.66 (.50) | 1.75 (.60) | -1.91 | .43 |
| C3 | 1.90 (.61) | 1.99 (.66) | -1.63 | .36 |
| C4 | 1.94 (.64) | 2.06 (.66) | -2.12* | .47 |
| FC3 | 1.78 (.57) | 1.89 (.62) | -2.14* | .48 |
| FC4 | 1.81 (.54) | 1.92 (.60) | -2.60* | .58 |
| FT7 | 1.61 (.57) | 1.68 (.63) | -.90 | .20 |
| FT8 | 1.54 (.54) | 1.62 (.60) | -1.33 | .30 |
| CP3 | 2.06 (.65) | 2.13 (.66) | -1.24 | .28 |
| CP4 | 2.11 (.75) | 2.21 (.71) | -1.62 | .36 |
| T7 | 1.45 (.59) | 1.56 (.55) | -1.37 | .31 |
| T8 | 1.42 (.62) | 1.50 (.65) | -.77 | .17 |
| P7 | 2.08 (.73) | 2.16 (.75) | -1.35 | .30 |
| P8 | 2.05 (.73) | 2.18 (.74) | -1.86 | .42 |
| P3 | 2.21 (.74) | 2.31 (.75) | -1.44 | .32 |
| P4 | 2.19 (.74) | 2.30 (.72) | -1.50 | .34 |
| O1 | 2.35 (.78) | 2.50 (.96) | -1.43 | .32 |
| O2 | 2.32 (.81) | 2.46 (.93) | -1.72 | .38 |

*indicates significance *p* < .05, and **indicates significance *p* < .01.
