# Supplementary material for: Unilateral Left-Hand Contractions Produce Widespread Depression of Cortical Activity after Their Execution
Source: PLoS One. 2015 Dec 28;10(12):e0145867. doi: 10.1371/journal.pone.0145867 (PMC4692494; doi:10.1371/journal.pone.0145867)
Supplement: S2 Table — (DOCX) [file pone.0145867.s008.docx]

**S2 Table.** *t*-scores and effect sizes for differences in alpha amplitudes between the phases before and after hand contractions for each electrode and each hand according to which hand-block was performed first.

| **Electrode Position** | **Left Hand-Block First** | | | | **Right Hand-Block First** | | | |
| --- | --- | --- | --- | --- | --- | --- | --- | --- |
|  | **Left Hand** | | **Right Hand** | | **Left Hand** | | **Right Hand** | |
|  | ***t*(9)** | ***d_z_*** | ***t*(9)** | ***d_z_*** | ***t*(9)** | ***d_z_*** | ***t*(9)** | ***d_z_*** |
| Fp1 | -2.78* | .88 | -.02 | .01 | -2.22 | .70 | -1.79 | .57 |
| Fp2 | -2.72* | .86 | .42 | .13 | -1.98 | .63 | -.84 | .27 |
| F3 | -3.12* | .99 | -.27 | .09 | -2.54* | .80 | -2.36* | .75 |
| F4 | -2.75* | .87 | -.04 | .01 | -2.37* | .75 | -1.77 | .56 |
| F7 | -2.38* | .75 | -1.12 | .35 | -2.32* | .73 | -1.84 | .58 |
| F8 | -1.30 | .41 | -.19 | .06 | -2.24* | .71 | -1.51 | .48 |
| C3 | -2.27* | .72 | -1.90 | .60 | -2.41* | .76 | -.19 | .06 |
| C4 | -3.10* | .98 | -.94 | .30 | -2.95* | .93 | -.98 | .31 |
| FC3 | -2.86* | .90 | -1.08 | .34 | -2.28* | .72 | -.31 | .10 |
| FC4 | -2.79* | .88 | -.98 | .31 | -2.72* | .86 | .04 | .01 |
| FT7 | -2.29* | .72 | -1.67 | .53 | -2.84* | .90 | -1.10 | .35 |
| FT8 | -2.51* | .79 | -.71 | .22 | -2.81* | .89 | -.67 | .21 |
| CP3 | -2.50* | .79 | -.78 | .25 | -2.73* | .86 | -.56 | .18 |
| CP4 | -3.57** | 1.13 | -1.44 | .46 | -2.95* | .93 | -1.66 | .52 |
| T7 | -1.48 | .47 | -1.39 | .44 | -3.43** | 1.08 | -1.00 | .32 |
| T8 | -1.68 | .53 | -1.86 | .59 | -3.51** | 1.11 | -1.57 | .50 |
| P7 | -2.09 | .66 | -.56 | .18 | -2.67* | .84 | -2.72* | .86 |
| P8 | -3.12* | .99 | -1.82 | .58 | -2.44* | .77 | -3.56** | 1.13 |
| P3 | -3.45** | 1.09 | -.43 | .14 | -2.61* | .83 | -1.30 | .41 |
| P4 | -3.61** | 1.14 | -1.74 | .55 | -2.89* | .91 | -1.83 | .58 |
| O1 | -3.19* | 1.01 | -1.05 | .33 | -1.82 | .58 | -2.52* | .80 |
| O2 | -3.34** | 1.06 | -1.73 | .55 | -2.31* | .73 | -2.59* | .82 |

*indicates significance *p* < .05, and **indicates significance *p* < .01.
